# Patient-specific factors associated with tumour motion in lung stereotactic body radiation therapy from real-time tumour tracking traces

**DOI:** 10.1016/j.phro.2025.100895

**Published:** 2025-12-16

**Authors:** Ashlesha Gill, Nicholas Bucknell, Mahsheed Sabet, Milad Mirzaei, Thomas Milan, Adriano Polpo, Pejman Rowshanfarzad

**Affiliations:** a School of Physics, Mathematics and Computing, The University of Western Australia, Crawley, WA 6009, Australia; b Department of Radiation Oncology, Sir Charles Gairdner Hospital, Nedlands, WA 6009, Australia; c Centre for Advanced Technologies in Cancer Research (CATCR), Nedlands, WA 6009, Australia

**Keywords:** Lung cancer, SBRT, Tumour motion, Personalised margins, CyberKnife

## Abstract

**Background and purpose:**

Respiratory motion is a major source of geometric uncertainty in lung stereotactic body radiation therapy (SBRT), necessitating individualized motion management. The study aimed to examine the lung tumour motion against patient- and tumour-specific factors.

**Materials and methods:**

Motion traces were obtained from 109 retrospective CyberKnife fiducial tracking lung treatments, recorded at 25 Hz through correlation of fiducial and external marker signals. Log files were collected and motion was quantified in the superior-inferior (SI), left–right (LR) and anterior-posterior (AP) directions. Each treatment delivery node (∼2700 per patient) was individually analysed. Clinical data included demographics, comorbidities, prior lung treatments, pulmonary function, and tumour location, size, and histology. Statistical analyses used multivariate and univariate approaches.

**Results:**

Tumour location strongly predicted SI motion, with lower lobes showing up to an 8.2 mm greater motion than upper lobes. Previous surgery or radiotherapy moderately reduced LR motion (–0.5 mm), while tumour diameter showed a weak positive association with LR motion (+0.02 mm per mm). Percentage predicted forced expiratory volume in one second (FEV_1_) showed moderate positive correlations with AP (+0.01 mm per %) and SI (+0.04 mm per %) motion. Body-mass index (BMI) weakly increased SI motion (+0.2 mm per kg/m^2^).

**Conclusions:**

Tumour location primarily determined SI motion, with additional increases linked to better pulmonary function and higher BMI. LR motion was greater in patients without prior lung treatment and with larger tumours, while greater AP motion occurred with better pulmonary function. Lung motion variation was quantified to support sub-millimetre SBRT precision.

## Highlights

• Tumour motion analysed with respect to patient factors using multivariate analysis.
• Lower lung lobes showed up to 8.2 mm more motion than upper lung lobes.
• Each 10 % decrease in forced expiratory volume in 1 s reduced motion by 0.4 mm.
• Prior lung surgery or radiotherapy reduced motion by 0.5 mm.
• Tumour diameter increased motion by 0.02 mm per unit.

## Graphical abstract

Body mass index increased motion by 0.2 mm per unit.

**Figure:**
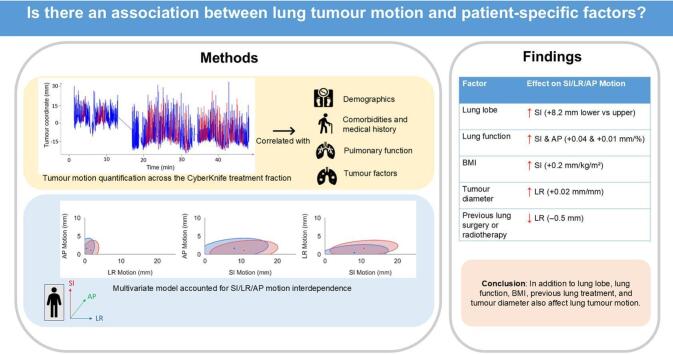

## 1 Introduction

Respiratory motion can introduce significant geometric uncertainties in the irradiation of lung tumours, making motion management crucial. More than half of the lung cancer patients present with smoking-related comorbidities such as chronic obstructive pulmonary disease (COPD) and cardiovascular diseases, making it critical to spare as much healthy tissue as possible [1], [2]. Stereotactic body radiation therapy (SBRT) is used to treat patients with early stage or oligometastatic cancer, offering improved local control and overall survival [3]. The most common method used for respiratory motion management is 4-dimensional (4D) computed tomography (CT) with an internal tumour volume [4]. To further mitigate respiratory motion, alternative techniques such as breath-hold, free breathing gating, or real-time tracking are commonly employed [4].

Although these methods represent substantial improvements over historical population-based margin approaches, they still have inherent uncertainties. 4D-CT has an inability to account for inter- and intrafraction variations in breathing, and studies have shown that 4D-CT may underpredict target motion in some cases [5], [6]. Breath-hold techniques require accurate timing and patient compliance [7], while free breathing gating and tracking techniques, though generally reliable, depend on the assumption that tumour motion can be accurately predicted from surrogates. Moreover, the availability of technical resources (e.g. staff, equipment) in radiotherapy department can be a limiting factor.

Understanding motion patterns in lung tumours may enable more individualized treatment margins. Few studies used real-time tumour tracking data to examine the effect of patient factors [8], [9], as most studies in this area have relied on 4D-CT scans [10], [11], [12], [13], [14], [15], [16], [17], [18]. While 4D-CT studies have extensively evaluated factors such as patient demographics, disease stage, tumour location, and size, aspects such as lung function and prior medical history have been rarely considered.

The aim of this study was to quantify lung tumour motion during SBRT and determine its association with routinely recorded patient- and tumour-specific characteristics. Using real-time tumour tracking traces from a robotic, image-guided SBRT system, motion amplitude was characterised for each beam-on segment and averaged across fractions to obtain a robust per-patient measure. This measure was analysed against a broad range of clinical factors using multivariate modelling.

## 2 Materials and methods

### 2.1 Tumour tracking traces

The study received ethics approval (Quality Activity No. 50342). It included a total of 109 primary early-stage lung cancer patients treated with the CyberKnife® M6^TM^ system (v. 11.2.x., Accuray Inc., USA) using fiducial tracking SBRT at Sir Charles Gairdner Hospital between 2014 and 2024. The data were deidentified before analysis. The CyberKnife Synchrony respiratory tracking system predicts internal tumour position by correlating fiducial markers with light emitting diode (LED) markers on the chest, continuously updating the model with intra-fraction X-ray imaging. Its prediction algorithm forecasts tumour movement 115 ms in advance based on the real-time LED coordinates [19]. Log files were extracted for all 109 patient plans and 525 treatment fractions. The Modeler.log file contained Synchrony-optimised tumour coordinates which were recorded at 25 Hz throughout each fraction. The accuracy of the log file coordinates were validated against measured tumour motion using the Synchrony quality assurance phantom (Accuray Inc., USA).

**Fig. 1:**
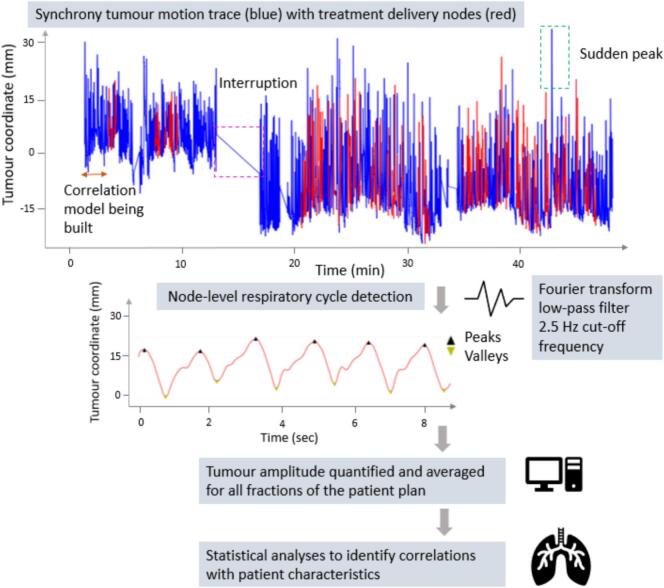
The Synchrony tumour motion trace from Modeler.log for each fraction was segmented into individual treatment node traces using ERsiData.log. After pre-processing, respiratory cycle detection was carried out using scipy.signal. The quantified tumour motion was correlated with patient characteristics.

Patient movement, coughing, deep breaths, apnoea, or technical machine errors could cause a deviation of the real-time tumour position from the expected position. Momentary deviations resolve if the breathing returns to normal, but in most cases, it leads to disruption of the correlation model, which then needs to be rebuilt. These events appeared in the motion data as interrupted time intervals and baseline shifts (Fig. 1). To account for this, each fractional motion trace was segregated into sub-traces that include only the treatment delivery nodes (robot positions during beam-on time), which are recorded in the ERsiData.log file (∼2719 ± 1361 nodes per plan) (Fig. 1). This ensured that the motion signals captured while the correlation model was still being prepared (e.g. before treatment delivery or after an interruption) were excluded from analysis.

### 2.2 Quantification of tumour amplitude

The system noise was removed using a Fast Fourier Transform-based low-pass filter with a cutoff frequency of 2.5 Hz [20]. The data from the first and last 0.5 s were excluded. Individual respiratory cycles were identified using the find_peaks function from the scipy.signal library (Fig. 1). To reliably distinguish true peaks from neighbouring fluctuations, a prominence threshold was set at 5 % of the total signal range, representing the minimum vertical distance expected between true peaks and nearby irregularities. Based on a minimum breathing cycle of 2 s, the minimum distance between consecutive peaks was set to 50 data points. The same criteria were applied to identify troughs. The peak-to-peak motion amplitude was quantified for each patient in the superior-inferior (SI), left–right (LR), and anterior-posterior (AP) directions, across different treatment nodes for all available fractions. These values were then used to calculate the average tumour motion for each patient in the SI, LR and AP directions.

### 2.3 Patient factors

Patient records for retrospective treatments were retrieved from the patient medical records to note patient characteristics, including demographics, tumour diameter, histology, tumour location (lung lobe), combined volume of lungs in the treatment planning system (TPS), body mass index (BMI), pre-bronchodilator pulmonary function test (PFT) parameters, lung comorbidities, previous surgery or radiotherapy, smoking history, and lung performance status. All PFT results were extracted from records within two years of the treatment date, BMI data within one year, and tumour diameter within eight months of treatment. The baseline characteristics are summarized in Table 1.

**Table 1:** Baseline characteristics; Abbreviations- n: Number of cases, %: percentage, BMI: body-mass index, SCC: squamous cell carcinoma, LCC: large cell carcinoma, LLL: left lower lobe, LUL: left upper lobe, RLL: right lower lobe, RUL: right upper lobe, RML: right middle lobe, FEV_1_: forced expiratory volume in 1 s, FVC: forced vital capacity, TLCO: transfer factor for carbon monoxide, COPD: chronic obstructive pulmonary disease, ILD: interstitial lung disease, RT: radiotherapy, ECOG: Eastern Cooperative Oncology Group performance status.

Characteristic	n	Median (Range)/n (%)
Age (yrs)	109	76 (55–90)
Gender	109	
*Male*		57 (52 %)
*Female*		52 (48 %)
BMI (kg/m^2^)	40	25.2 (17.6–44.4)
Histology	91	
*Adenocarcinoma*		66 (73 %)
*SCC*		23 (25 %)
*LCC*		2 (2 %)
Tumour diameter (mm)	83	17.0 (7.0–57.8)
Lung lobe	107	
*LLL*		18 (17 %)
*LUL*		33 (31 %)
*RLL*		21 (20 %)
*RUL*		29 (27 %)
*RML*		6 (5 %)
TPS combined lungs volume (L)	62	3.2 (1.6–5.9)
Measured FEV_1_ (L)	102	1.8 (0.4–3.2)
% Predicted FEV_1_ (%)	101	73 (21–153)
Measured FVC (L)	100	2.8 (1.1–5.1)
% Predicted FVC (%)	97	87 (35–152)
measured FEV_1_/FVC	100	0.6 (0.2–0.9)
Measured TLCO (ml/min/mmHg)	70	12.7 (5.4–26.0)
% Predicted TLCO (%)	81	60 (24–113)
COPD	98	
*Yes*		66 (67 %)
*No*		32 (33 %)
Asthma	64	
*Yes*		12 (19 %)
*No*		52 (81 %)
ILD	58	
*Yes*		5 (9 %)
*No*		53 (91 %)
Previous lung surgery/RT	95	
*Yes*		22 (23 %)
*No*		73 (77 %)
Pack-years (pack-year)	85	40 (0–152)
Years smoked (yrs)	62	40 (0–70)
ECOG	86	
*0*		21 (24 %)
*1*		42 (49 %)
*≥2*		23 (27 %)

### 2.4 Statistical analysis

Statistical analyses were conducted to explore the relationship between patient characteristics and tumour motion in each direction. Since the data were not normally distributed, non-parametric tests were used for univariate analyses. Data were merged using unique identifiers for each patient and treatment plan. Spearman’s rank correlation was applied for numerical variables, with scatterplots created for visualization. The Mann-Whitney *U* test was applied to categorical variables with two categories, while the Kruskal-Wallis H test was used for variables with more than two categories, followed by Dunn’s test with Bonferroni correction for pairwise comparisons. Where applicable, additional subgroup analyses were conducted. Trends were visually assessed using boxplots. To assess the combined influence of multiple patient factors across the three motion axes (SI, LR, and AP), a multivariate linear regression model (multivariate ordinary least squares) was fitted. Covariates included patient factors showing the strongest correlations with tumour motion in preliminary analyses. Multivariate significance for each predictor was assessed using Pillai’s Trace statistic, which provides a robust test in the presence of non-normal residuals or unequal group variances. Heteroskedasticity-consistent (HC3)-robust standard errors were applied for each motion axis to mitigate heteroskedasticity and obtain regression coefficients (β) with 95 % confidence intervals. All tests were two-tailed, with statistical significance level set at p < 0.05. All analyses were carried out in Python 3 using the scipy.stats and statsmodels libraries.

## 3 Results

### 3.1 Demographics and medical history

Age at the time of treatment showed a weak positive correlation with tumour motion in the AP direction (Spearman’s ρ = 0.2, p = 0.03). BMI also exhibited a weak association with tumour motion in the AP direction, but this did not reach statistical significance (Spearman’s ρ = 0.3, p = 0.09). No association was identified for tumour motion and gender or performance status. Patients with a history of lung surgery and radiotherapy demonstrated lower tumour motion in the LR direction compared to those without (Mann-Whitney U = 541, p = 0.02). Pack-years of smoking demonstrated a weak negative correlation with AP tumour motion, though this was not statistically significant (Spearman’s ρ = −0.2, p = 0.11). No association was observed between years smoked and tumour motion.

### 3.2 Impaired lung function

A weak positive correlation was observed between percentage predicted forced expiratory volume in 1 s (FEV_1_) and tumour motion in the AP direction (Spearman’s ρ = 0.2, p = 0.08). Similarly, FEV_1_/forced vital capacity (FVC) showed a weak association with AP tumour motion, but this did not reach statistical significance (Spearman’s ρ = 0.2, p = 0.06). No significant correlations were found for measured FEV_1_, measured FVC, measured transfer factor for carbon monoxide (TLCO), percentage predicted FVC, or percentage predicted TLCO. Lung volume, derived from TPS contours of the combined lungs in the exhale phase, was not associated with tumour motion. Patients with COPD exhibited slightly higher AP tumour motions compared to those without, but this difference was not statistically significant (Mann-Whitney U = 1262, p = 0.12). Similarly, patients with asthma demonstrated lower SI tumour motions compared to those without; however, this difference also failed to reach statistical significance (Mann-Whitney U = 400, p = 0.13). For patients with documented interstitial lung disease (ILD), the small sample size precluded reliable statistical inferences.

### 3.3 Tumour and motion characteristics

The tumour motion, averaged across all patients, was 5.7 ± 5.4 mm in the SI direction, 1.1 ± 0.9 mm in the LR direction, and 1.7 ± 1.2 mm in the AP direction (mean ± standard deviation). Table 2 summarizes the variation in tumour motion across different categorical patient factors.

**Table 2:** Results from Mann-Whitney *U* test and Kruskal-Wallis H test, including tumour motion median and range values; Abbreviations- SI: superior-inferior, LR: left–right, AP: anterior-posterior, SCC: squamous cell carcinoma, LCC: large cell carcinoma, LLL: left lower lobe, LUL: left upper lobe, RLL: right lower lobe, RUL: right upper lobe, RML: right middle lobe, COPD: chronic obstructive pulmonary disease, ILD: interstitial lung disease, RT: radiotherapy, ECOG: Eastern Cooperative Oncology Group performance status; Footnotes- ^†^: Mann-Whitney *U* test, ^‡^: Kruskal-Wallis H test, *: statistically significant (p < 0.05).

Characteristic	Category	Tumour motion, mm [median (range)]			P value		
		SI	LR	AP	SI	LR	AP
Gender^†^	Male	3.2 (0.2–22.8)	0.9 (0.1–5.3)	1.5 (0.1–5.9)	0.38	0.62	0.37
	Female	4.5 (0.2–18.8)	0.9 (0.3–3.7)	1.5 (0.4–5.5)			
Histology^‡^	Adenocarcinoma	4.1 (0.2–22.8)	0.9 (0.2–3.3)	1.4 (0.1–5.9)	0.53	0.86	0.74
	SCC	3.4 (0.3–13.7)	0.8 (0.1–5.3)	1.5 (0.3–3.1)			
	LCC	5.3 (4.1–6.6)	1.4 (0.6–2.2)	1.8 (1.7–1.8)			
Lung lobe^‡^	LLL	7.7 (3.1–22.3)	0.6 (0.3–3.1)	1.3 (0.5–4.4)	<0.001*	0.70	0.28
	LUL	2.1 (0.3–12.1)	0.9 (0.1–5.3)	1.5 (0.1–5.9)			
	RLL	12.4 (2.7–22.8)	1.0 (0.3–3.4)	1.9 (0.3–5.2)			
	RUL	2.4 (0.2–13.4)	0.8 (0.2–2.4)	1.4 (0.5–5.5)			
	RML	3.1 (0.2–11.5)	0.7 (0.4–1.6)	1.1 (0.1–2.4)			
COPD^†^	Yes	3.5 (0.2–18.8)	0.8 (0.1–5.3)	1.4 (0.1–5.9)	0.68	0.84	0.12
	No	3.6 (0.2–22.8)	1.0 (0.3–3.3)	1.8 (0.2–5.7)			
Asthma^†^	Yes	2.5 (0.7–13.3)	0.8 (0.1–5.3)	1.6 (1.1–2.7)	0.13	0.99	0.58
	No	4.5 (0.2–22.8)	0.8 (0.2–3.4)	1.5 (0.2–5.7)			
ILD^†^	Yes	9.9 (2.4–13.0)	1.0 (0.3–3.1)	1.6 (0.5–3.1)	0.16	1.00	0.87
	No	4.1 (0.2–22.8)	0.8 (0.2–3.4)	1.7 (0.2–5.7)			
Previous lung surgery/RT^†^	Yes	3.5 (0.2–22.8)	0.6 (0.1–2.2)	1.4 (0.1–4.4)	0.23	0.02*	0.68
	No	4.4 (0.2–22.3)	0.9 (0.2–5.3)	1.5 (0.1–5.9)			
ECOG^‡^	0	3.5 (0.2–15.1)	0.9 (0.1–2.8)	1.4 (0.1–5.9)	0.53	0.74	0.54
	1	4.0 (0.2–22.8)	0.8 (0.2–5.3)	1.5 (0.1–5.7)			
	≥2	4.7 (0.6–22.3)	1.1 (0.2–3.3)	1.8 (0.4–5.2)			

The tumour diameter showed a weak positive correlation with tumour motion in the LR direction (Spearman’s ρ = 0.3, p = 0.01). No association was observed between lesion histology and tumour motion.

Tumour motions in the SI direction varied substantially across lung lobes (Kruskal-Wallis H = 51, p < 0.001), with lower lobes (left lower lobe (LLL), right lower lobe (RLL)) showing greater motion than upper (left upper lobe (LUL), right upper lobe (RUL)) and right middle lobe (RML). Dunn’s test revealed significant differences for LLL vs. LUL, RLL vs. RUL, RLL vs. LUL, and LLL vs. RUL (all p < 0.001). The RML vs. RLL difference was borderline (p = 0.03).

Since lung lobe had the strongest effect, an additional analysis was performed, separately assessing tumours in upper/middle lobes (n = 68) and lower lobes (n = 39).

In the upper/ middle lobes, age significantly but weakly correlated with AP tumour motion (ρ = 0.3, p = 0.01), and percentage predicted FEV_1_ also showed a significant weak correlation with AP tumour motion (ρ = 0.3, p = 0.02). Patients with a history of previous lung surgery or radiotherapy exhibited lower LR motion (U = 187, p = 0.02). The correlation between tumour diameter and LR tumour motion was not statistically significant (p = 0.093).

In the lower lobes, tumour diameter had a significant moderate correlation with LR tumour motion (ρ = 0.4, p = 0.02).The FEV_1_/FVC ratio (ρ = 0.5, p = 0.002) had a moderate positive correlation and measured FVC (ρ = -0.3, p = 0.04) had a weak negative correlation with SI tumour motion. Previous lung surgery or radiotherapy appeared to correlate with SI tumour motion (p = 0.09), but this result did not reach statistical significance.

### 3.4 Multivariate analysis

**Fig. 2:**
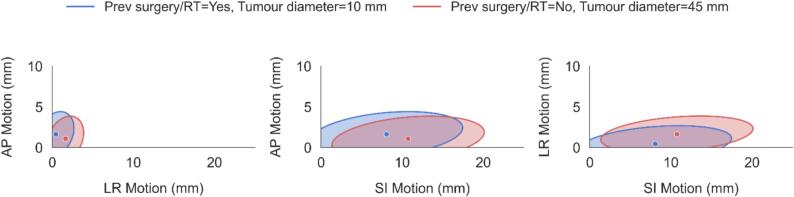
Predicted 95 % motion ellipses from the multivariate model’s residual covariance (Σ), illustrating the joint distribution of SI, LR, and AP motion. Tumour diameter and prior lung surgery/RT status were varied, while Lung lobe (LLL), BMI (30 kg/m^2^), and percentage predicted FEV_1_ (70) were held constant.

Moderate residual correlations were observed between the motion axes (ρ(SI–LR) = 0.3, ρ(SI–AP) = 0.3, ρ(LR–AP) = 0.3), confirming interdependence among the dependent variables and supporting the use of a multivariate framework. Fig. 2 further illustrates this interrelationship, showing that factors influencing LR motion (e.g., tumour diameter and previous surgery or radiotherapy) also affected motion in the SI and AP directions.

On multivariate testing using Pillai’s Trace, lung lobe (F = 5.4, p < 0.001), tumour diameter (F = 3.1, p = 0.03), percentage predicted FEV_1_ (F = 5.0, p = 0.003), and BMI (F = 2.9, p = 0.04) showed significant overall effects on 3D tumour motion amplitudes across the SI, LR, and AP directions.

**Fig. 3:**
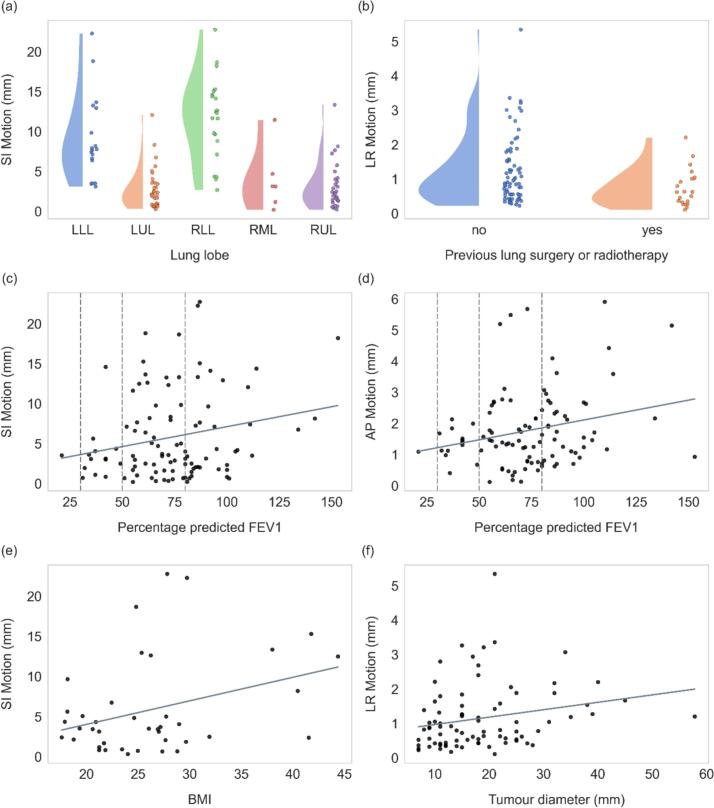
Association between: (a) lung lobe and SI tumour motion; (b) previous lung surgery or radiotherapy and LR motion; (c) percentage predicted FEV_1_ and SI motion; (d) percentage predicted FEV_1_ and AP motion (dashed vertical lines represent the GOLD spirometry criteria for COPD severity: >80 % (normal), 50–80 % (mild COPD), 30–50 % (severe COPD), and <30 % (very severe COPD) [21]); (e) BMI and SI motion; and (f) tumour diameter and LR motion.

Fig. 3 and Table 3 summarize the per-axis analyses. Tumour location was a strong predictor of increased SI motion, with the following results: LLL vs LUL (β = 5.49, 95 % CI [2.79 to 8.19], p < 0.001), RLL vs RUL (β = 8.18, 95 % CI [5.80 to 10.56], p < 0.001), and RLL vs RML (β = 6.12, 95 % CI [1.92 to 10.32], p = 0.004). Previous surgery or radiotherapy was associated with a moderate reduction in LR motion (β = -0.51, 95 % CI [-0.83 to −0.19], p = 0.002). Tumour diameter showed a weak positive association with LR motion (β = 0.02, 95 % CI [0.00 to 0.04], p = 0.045). These findings were consistent with univariate tests. For percentage predicted FEV_1_, a moderate positive association was observed with AP motion (β = 0.01, 95 % CI [0.00 to 0.03], p = 0.03), and an additional moderate association was found with SI motion (β = 0.04, 95 % CI [0.01 to 0.07], p = 0.02). BMI also emerged as a weak but significant predictor of SI motion (β = 0.22, 95 % CI [0.03 to 0.42], p = 0.03).

**Table 3:** Results from Multivariate linear regression (β coefficients with heteroskedasticity-consistent (HC3) 95 % confidence intervals). Abbreviations- SI: superior-inferior, LR: left–right, AP: anterior-posterior, LLL: left lower lobe, LUL: left upper lobe, RLL: right lower lobe, RUL: right upper lobe, RML: right middle lobe, RT: radiotherapy, FEV_1_: forced expiratory volume in 1 s, BMI: body-mass index; Footnotes- *: statistically significant (p < 0.05).

Characteristic	SI β (95 % CI)	P value	LR β (95 % CI)	P value	AP β (95 % CI)	P value
LLL vs LUL	5.49 (2.79, 8.19)	<0.001*	−0.33 (−0.90, 0.23)	0.24	−0.41 (−1.09, 0.28)	0.24
RLL vs RUL	8.18 (5.80, 10.56)	<0.001*	0.30 (−0.21, 0.81)	0.24	0.21 (−0.48, 0.90)	0.55
RLL vs RML	6.12 (1.92, 10.32)	0.004*	0.30 (−0.34, 0.95)	0.35	0.68 (−0.14, 1.50)	0.10
Previous lung surgery/RT (yes vs no)	−1.91 (−3.98, 0.16)	0.07	−0.51 (−0.83, −0.19)	0.002*	−0.31 (−0.82, 0.21)	0.25
% Predicted FEV_1_ (%)	0.04 (0.01, 0.07)	0.02*	−0.00 (−0.01, 0.00)	0.31	0.01 (0.00, 0.03)	0.03*
BMI (kg/m^2^)	0.22 (0.03, 0.42)	0.03*	−0.00 (−0.05, 0.05)	1.00	0.05 (−0.02, 0.12)	0.14
Tumour diameter (mm)	0.02 (−0.07, 0.12)	0.66	0.02 (0.00, 0.04)	0.045*	−0.02 (−0.05, 0.00)	0.07

## 4 Discussion

Tumour motion was evaluated with respect to clinical factors in 109 retrospective lung cancer cases. Depending on the direction of motion, significant associations were identified with tumour location and diameter, BMI, lung function, and history of lung surgery or radiotherapy.

Lung lobe remained a strong predictor of SI tumour motion, consistent with the findings of previous studies [12], [18], [22]. While tumours in the upper and middle lobe exhibited significantly less motion than lower lobe tumours, 12 (18 %) of upper/middle lobe tumours still showed average motion greater than 5 mm, and in some cases >10 mm. Onodera et al. [8] reported that the three-dimensional maximal motion could exceed 20 mm in selected upper lobe cases, suggesting that tumour location alone may not fully determine tumour displacement.

Patients with a history of lung surgery or radiotherapy had significantly reduced LR tumour motion, which was also significant when analysis was restricted to upper-lobe tumours. The effect is likely due to the fibrosis following treatment – this aligns with a previous study, which reported that tumours in the upper lobes with fibrosis exhibited reduced motion compared to those without fibrosis [8]. Multivariate analysis indicated that patients with previous surgery or radiotherapy had on average, 0.5 mm lower LR motion, with reductions of up to approximately 0.8 mm.

The relationship between tumour motion and tumour diameter was inconclusive. Although a significant positive correlation with LR motion was observed, the majority of tumours with relatively larger motion had a diameter of less than 20 mm (4.2 cm^3^). This may reflect confounding influences, as multivariate analysis confirmed only a weak positive correlation. Previous studies have reported dominant motion for small tumours (up to 20 cm^3^) across all directions [14], larger AP motion for larger tumours (>45 cm^3^) in upper/middle lobes [12], or no clear dependence on tumour size [9], [17], [22].

Older patients had slightly larger tumour motion in the AP direction as per the univariate test. In some patients >70 years of age, the AP tumour motion was found be higher than 5 mm. Adamczyk et al. [14] observed a similar correlation between age and tumour motion in all three directions. In the present study, the association did not remain significant in the multivariate model.

A reduction in AP and SI tumour motion was observed with lower percentage predicted FEV_1_ (increased COPD severity). Multivariate analysis showed that each 10 % reduction in FEV_1_ was associated with an approximate 0.1 mm decrease in AP motion and 0.4 mm decrease in SI motion. In a sub-analysis of lower lobe cases, a statistically significant reduction in SI tumour motion was observed with lower FEV_1_/FVC and statistically significant increase in SI tumour motion with measured FVC. However, stratification into upper and lower lobes considerably reduced the sample size, so the sub-analysis requires further validation. Some studies reported in the literature have focused on the impact of lung function on diaphragm motion rather than its effect on tumour motion [23]. Bakker et al. [24] found a direct association between COPD severity and a reduced diaphragm index, indicating diaphragm impairment. Additionally, abnormal hemidiaphragmatic motion in COPD patients has been documented, with the ventral portion moving downward and the dorsal portion moving upward [25]. Onodera et al. [8] evaluated the impact of emphysema (using % low attenuation volume on CT scan and FEV_1_/FVC) on tumour motion but found no correlation.

BMI showed a weak but statistically significant association with SI motion, with each 1 kg/m^2^ increase corresponding to approximately 0.2 mm greater motion. This modest effect may reflect enhanced diaphragmatic excursion in individuals with higher body mass [26], [27].

All other factors, including gender, histology, lung volume, asthma, ILD, smoking history, and performance status, showed no significant effect on tumour motion.

The present study is exploratory in nature and requires further validation before it can be used to inform clinical practice. Some of the observed associations, particularly in the AP and LR directions, were near the threshold of statistical significance. This may reflect the influence of inter- and intra-fractional motion variability, additional unmeasured factors, or residual noise. Due to insufficient sample size, the multivariate analysis couldn’t be performed for upper and lower lobes separately. Future studies with larger and more representative samples are recommended to replicate the analysis and further investigate motion patterns in both upper/middle and lower lobes.

By analysing tumour motion data acquired throughout the treatment fraction, this study provided a more representative measure of average motion than 4D-CT studies. Clinically recorded parameters were evaluated using non-parametric tests and multivariate linear regression. The multivariate approach accounted for both the combined influence of patient factors and the interdependence of motion across the three axes—an aspect acknowledged but rarely incorporated in previous analyses. Several findings aligned with prior studies, but notably, this is the first to demonstrate a direct association between tumour motion and lung function or prior surgery/radiotherapy. Although many significant motion reductions observed in this study were limited to a few millimetres, they may still be clinically meaningful given the sub-millimetre precision required for lung SBRT. Such insights could support the development of personalised treatment margins, offering significant benefits to institutions that lack access to advanced motion management technologies for lung radiotherapy.

In conclusion, SI tumour motion was primarily predicted by lung lobe, but was also significantly reduced in patients with poor lung function and increased with higher BMI. Although LR motion was generally small in magnitude, a history of lung surgery or radiotherapy was moderately associated with reduced motion, while tumour diameter showed a weak positive association. In the AP direction, reduced tumour motion was observed with poor lung function.

## CRediT authorship contribution statement

**Ashlesha Gill:** Conceptualization, Data curation, Formal analysis, Funding acquisition, Investigation, Methodology, Software, Validation, Visualization, Writing – original draft. **Nicholas Bucknell:** Conceptualization, Methodology, Resources, Supervision, Funding acquisition, Writing – review & editing. **Mahsheed Sabet:** Conceptualization, Methodology, Resources, Supervision, Funding acquisition, Writing – review & editing. **Milad Mirzaei:** Conceptualization, Methodology, Resources, Supervision, Funding acquisition, Writing – review & editing. **Thomas Milan:** Resources, Software, Writing – review & editing. **Adriano Polpo:** Formal analysis, Resources, Writing – review & editing. **Pejman Rowshanfarzad:** Conceptualization, Methodology, Project administration, Resources, Supervision, Funding acquisition, Writing – review & editing.

## Funding

The research was funded through Charlie’s Foundation of Research Discovery Grant DGP24-25_10 alongside the University of Western Australia Postgraduate Award and International Fee Scholarship. The funding sources had no involvement in the study design, data analysis, report writing, or any other aspects of the work.

## Declaration of competing interest

The authors declare that they have no known competing financial interests or personal relationships that could have appeared to influence the work reported in this paper.
